# Information-Theoretical Criteria for Characterizing the Earliness of Time-Series Data

**DOI:** 10.3390/e22010049

**Published:** 2019-12-30

**Authors:** Mariano Lemus, João P. Beirão, Nikola Paunković, Alexandra M. Carvalho, Paulo Mateus

**Affiliations:** 1 Instituto de Telecomunicações, 1049-001 Lisboa, Portugal; 2 Instituto Superior Técnico, Universidade de Lisboa, 1049-001 Lisboa, Portugal

**Keywords:** Akaike information criterion, minimum description length, time-series charaterization

## Abstract

Biomedical signals constitute time-series that sustain machine learning techniques to achieve classification. These signals are complex with measurements of several features over, eventually, an extended period. Characterizing whether the data can anticipate prediction is an essential task in time-series mining. The ability to obtain information in advance by having early knowledge about a specific event may be of great utility in many areas. Early classification arises as an extension of the time-series classification problem, given the need to obtain a reliable prediction as soon as possible. In this work, we propose an information-theoretic method, named Multivariate Correlations for Early Classification (MCEC), to characterize the early classification opportunity of a time-series. Experimental validation is performed on synthetic and benchmark data, confirming the ability of the MCEC algorithm to perform a trade-off between accuracy and earliness in a wide-spectrum of time-series data, such as those collected from sensors, images, spectrographs, and electrocardiograms.

## 1. Introduction

A time-series (TS) consists of measurements or observations acquired and organized sequentially over time. In this context, one or multiple variables may be examined, being the first-named univariate time-series (UTS) and the second multivariate time-series (MTS). Several data mining application areas deliver this sort of data, such as medicine, economy, meteorology, and marketing. Standard TS classification involves using temporal data for constructing a classifier, which can predict the class label of a new given TS, with satisfactory accuracy.

Early classification (EC) is an extension of the TS classification problem, and it arises in scenarios where the anticipation of the prediction is beneficial. This matter has recently been a relevant subject of study, due to its several time-sensitive applications. For instance, a medical study [1] described how clinical data revealed that infants diagnosed with sepsis disease suffered from an unusual heartbeat twenty-four hours before the diagnosis. In this case, supervising the TS data of the infant’s heartbeat and being able to classify it in advance may lead to effective early diagnosis and treatment.

The work from Xing et al. [2] was one of the first to formulate the problem of EC, proposing to unveil a timestamp from which the information of the TS from that point on is irrelevant. As stated by Xing et al. [3], it is vital to distinguish EC from classic TS prediction, where the goal is to forecast values given the whole TS. EC of temporal data consists of anticipating the classification by using only a portion of the available information, without compromising the prediction quality. There are two essential requirements for an early classifier: being able to designate the earliest time location (timestamp) of accurate classification, and ensuring an accuracy close to the case of using the full-length TS.

Several methods addressing the early classification problem have been proposed in the last years [2,4,5,6,7,8]. These approaches require a learning stage followed by a classification step, being able to assign a class label to a single incomplete time-series. Notwithstanding the significant advances in EC, there are temporal datasets for which it is intrinsically hard to perform early predictions, due to phenomenons like high conductance of the underlying Markov model, or cumulative errors throughout the time measurements.

In contrast, we propose a dataset investigation, where the information from the entire collection of time-series (and not only a single TS), as well as their respective class labels, is the subject of study. Our approach explores the knowledge contained in the data via the temporal correlation of the variables. In general, testing all possible timestamps without external information would require an expensive, and in some cases, computationally unfeasible, cross-validation search. We propose to use information-theoretical criteria to perform this analysis efficiently throughout the whole data.

Overall, the main contribution of this work is applying information-theoretic criteria for examining the EC opportunity in a dataset containing univariate or multivariate TS, extracting a global timestamp (for the overall data) from which accurate prediction might be performed. An EC method, like those mentioned before, can then be applied, with a priori knowledge of the EC opportunity in the data.

We assessed the merits of the proposed method in a wide-spectrum of time-series data, such as those collected from sensors, images, spectrographs, and electrocardiograms. In particular, we found that for a collection of time-series that traces the electrical activity recorded during one heartbeat, the first 30% of the signal contains enough information to discriminate between a normal heartbeat and a myocardial infarction, leveraging the development of novel methods to focus in this fraction of the signal. An implementation of the proposed criteria, through a procedure called Multivariate Correlations for Early Classification (MCEC), is made freely available to the comunity at https://joaopbeirao.github.io/MCECalgorithm/.

The paper is organized as follows. In Section 2, we review some basic concepts of Bayesian networks and multivariate correlation. In Section 3, we present our EC opportunity algorithm, followed by experimental results over benchmark data; results in synthetic data are left for the Appendices. Finally, we draw some conclusions and discuss future work.

## 2. Background

We start by introducing Bayesian networks as they have a well-established framework to measure model complexity. In this regard, we choose two criteria: minimum description length and Akaike information criterion. Finally, we present multivariate correlations to assess data earliness by deriving the dependencies among the variables through a concrete Bayesian network calibrated with the adopted criteria.

### 2.1. Bayesian Networks

Probabilistic graphical models attempt to describe the behaviour of complex systems using a graph-based framework for representing the probability distributions [9]. Bayesian networks (BNs) are probabilistic graphical models for describing complex domains, and they can be used to represent the information about an uncertain system [10,11,12]. The BN representation consists of a directed acyclic graph *G*, characterized by a set of nodes $N=\left\{X_{1},X_{2},\ldots ,X_{n}\right\}$ and a set of directed edges *E*. Considering a G=(N,E), each node (vertex) corresponds to a random variable $X_{i}$, and the edges (arrows), that connect the nodes in a specific direction, describe the probabilistic dependencies between the random variables. For each node $X_{i}$, two sets can be defined: the set of parents $\Pi_{X_{i}}$ and the set of non-descendants $\Phi_{X_{i}}$. The structure of a BN is based on the assumption that each node $X_{i}$ is conditionally independent of $\Phi_{X_{i}}$, provided that the values of the variable in $\Pi_{X_{i}}$ are known. The group of local probability models, representing the dependence of each variable $X_{i}$ on $\Pi_{X_{i}}$, specifies the parameters for describing the network structure. These form the set of conditional probability distributions

$$\Theta ={\left\{\theta_{X_{i}|\Pi_{X_{i}}}\right\}}_{i\in \left\{1,\ldots ,n\right\}},$$

where $\theta_{X_{i}|\Pi_{X_{i}}}=P\left(X_{i}=x_{i}|\Pi_{X_{i}}=\omega_{i}\right)$, associated to each node $X_{i}$ and conditioned on $\Pi_{X_{i}}$.

A BN B=(G,Θ) is comprised of the direct acyclic graph structure *G* together with the set of parameters Θ. The joint probability distribution defined by this representation is calculated as:

(1) $$P_{B}\left(X_{1},\ldots ,X_{n}\right)\;=\;\prod_{i=1}^{n}P_{B}\left(X_{i}|\Pi_{X_{i}}\right)\;=\;\prod_{i=1}^{n}\theta_{X_{i}|\Pi_{X_{i}}}.$$

For a given a multinomial dataset *D* of size *N*, the problem of learning a BN consists of designing the B=(G,Θ) that best represents *D*, according to some scoring function [13]. This scoring criterion corresponds to the search guide for evaluating the effectiveness of the network in representing the data. Moreover, when the structure of the network is fixed, the parameters Θ that optimize the likelihood, for a given dataset, are those described by the observed frequency estimates:

(2) $${\hat{P}}_{B}\left(X_{i}=x_{i}|\Pi_{X_{i}}=\omega_{i}\right)=\frac{|D_{x_{i},\omega_{i}}|}{|D_{\omega_{i}}|},$$

for which $|D_{x_{i},\omega_{i}}|$ represents the number of instances in *D*, where $X_{i}$ takes the value $x_{i}$, and its parents $\Pi_{X_{i}}$ take the value $\omega_{i}$. Similarly, $|D_{\omega_{i}}|$ denotes the number of instances in *D*, where $\Pi_{X_{i}}$ takes the value $\omega_{i}$.

The Minimum Description Length (MDL) principle is known as an Occam’s razor approach to select, for a given dataset, the best fitting model and its parameters [14]. It is a widely used metric which states that, for a certain data and a number of alternative models, the best option corresponds to the simplest model [15,16,17]. In the problem of learning a BN, the Bayesian Information Criterion (BIC) is known as the MDL score. It is concerned with analysing the trade-off between the Log-Likelihood (LL) of the dataset *D* (the effectiveness of the fit to the data) and the complexity of the model. This scoring function [13] is defined as

(3) $$MDL\left(D|B\right)=-LL\left(D|B\right)+\frac{\log_{2}N}{2}\left|B\right|,$$

where *N* corresponds to the size of the data, and |B| represents the model dimension (number of parameters in B). The LL term quantifies the amount of information required to describe the dataset *D*, using B. Conversely, the penalty term measures the amount of information needed to encode the model B. The overall goal of the MDL score is to elicit the model that most effectively fits the dataset, provided that its complexity is as low as possible, in this way avoiding overfitting.

Similarly to the MDL scoring function, the Akaike Information Criterion (AIC) [18] corresponds to a measure of the quality of statistical models for describing a given dataset. In the problem of learning a BN, the difference between MDL and AIC is associated to the penalty applied to the number of parameters |B|. The AIC scoring function [13] can be defined as:

(4) AIC(D∣B)=−LL(D∣B)+|B|.

In Equation (3), the second term quantifies the amount of information required to encode the model B, where each parameter in the set Θ is considered to use $\frac{1}{2}\log_{2}N$ bits. Conversely, in Equation (4) each parameter of Θ is considered to use 1 bit. This means that the penalization on the number of independent parameters is stronger in the MDL scoring function than in the AIC score. Likewise for the MDL score, the best model corresponds to the one that minimizes Equation (4).

Literature complies with the fact that these two criteria demonstrate different properties for model selection and that they are appropriate according to specific conditions [19,20,21]. According to Vrieze [21], MDL is considered to be consistent in selecting the true model, with probability close to one, given that the true model is in the set of candidate models. On the other hand, if the true model is not in the set of alternatives, AIC is considered to be effective, since it selects the model that minimizes the mean squared error of the estimation. However, both criteria are unsuitable for dealing with low dimensional datasets for which the number of instances is close to the number of parameters to estimate [22].

### 2.2. Multivariate Correlations

We briefly introduce multivariate correlations to derive a concrete Bayesian network from which we deduce model complexity. From a statistical point of view, the concept of correlation between variables attempts to measure the relationships and dependencies among them. The knowledge of how the variables are related, as well as of what inferences can be made about their causal relationships, is useful for drawing conclusions about potential predictive relationships to be analyzed and exploited.

For a finite set of discrete random variables $S={\left\{X_{i}\right\}}_{i=1,\ldots ,n}$, with a joint probability distribution $P_{S}\left(X_{1},\ldots ,X_{n}\right)$, the total correlations between those variables can be defined as [23]:

(5) $$I\left(X_{1},\ldots ,X_{n}\right)=\sum_{i=1}^{n}H\left(X_{i}\right)-H\left(X_{1},\ldots ,X_{n}\right).$$

In this case, the mutual information measures the dependencies among the variables, i.e., the amount of information that these quantities give about each other.

Let a structural relation *R* be a subset of the system *S*. Its joint probability distribution corresponds to the marginal distribution from *S*

(6) $$P_{R}\left(X_{R_{1}},\ldots ,X_{R_{k}}\right)=\sum_{X_{i}\notin R}P_{S}\left(X_{1},\ldots ,X_{n}\right),$$

where *k* is the number of elements in *R*.

Following the maximum entropy principle applied to fixed marginals [24], we say that a structure associated to the system *S* with underlying joint probability $P_{S}$ is a pair $\left(S,P_{S}\right)$, where $S={\left\{R_{j}\right\}}_{j=1,\ldots ,k}$ is a collection of structural relations and $P_{S}$ is (another) joint probability distribution over *S*, such that:

1. No $R_{i}\in S$ is contained in another $R_{j}\in S$ (or in other words, $\forall_{i\neq j}\;R_{i}⊈R_{j}$);
2. Every $X_{i}\in S$ is included in at least one $R_{j}\in S$;
3. $P_{S}$ is the solution to the optimization problem:
  $$\max_{P\in P}\;\;H\left(P\right)\;\;s.t.\;\;\sum_{X_{i}\notin R_{j}}\;P_{S}\left(X_{1},\ldots ,X_{n}\right)\;=\;\sum_{X_{i}\notin R_{j}}\;P_{S}\left(X_{1},\ldots ,X_{n}\right),\forall R_{j}\in S,$$
  where P is the set of probability distributions of the variables from *S*.

For example, from the set of discrete random variables $S=\left\{X_{1},X_{2},X_{3},X_{4}\right\}$, some admissible structures S are $\left\{\left\{X_{1},X_{2},X_{3}\right\},\left\{X_{4}\right\}\right\}$, $\left\{\left\{X_{1},X_{2}\right\},\left\{X_{3},X_{4}\right\}\right\}$ or $\left\{\left\{X_{1},X_{2}\right\},\left\{X_{1},X_{4}\right\},\left\{X_{2},X_{3},X_{4}\right\}\right\}$. On the other hand, the structure

$$S=\left\{\left\{X_{1},X_{2},X_{3}\right\},\left\{X_{1},X_{3}\right\},\left\{X_{4}\right\}\right\}$$

is not an acceptable structure since the relation between $X_{1}$ and $X_{3}$ is included in two structural relations, which violates the first condition. Similarly, $S=\left\{\left\{X_{1},X_{2}\right\},\left\{X_{2},X_{4}\right\}\right\}$ does not consist of a proper structure because the variable $X_{3}\in S$ is not part of any structural relation from S, as required by the second condition.

For a given system $S={\left\{X_{i}\right\}}_{i=1,\ldots ,n}$ and an associated set of structural relations $S={\left\{R_{j}\right\}}_{j=1,\ldots ,k}$, the mutual information I(S) represents the maximum amount of information that the variables $X_{i}$ from *S* provide about each other. On the other hand, I(S) quantifies the information described by the correlations only inside the structural relations $R_{j}$. The difference I(S)−I(S) measures the knowledge of the dependencies and relationships between the variables of *S* that are not included in the relations that compose S. From Equation (5), this value can be described as a difference of entropies:

(7) $$\begin{array}{c} I\left(S\right)-I\left(S\right)=\sum_{i=1}^{n}H\left(X_{i}\right)-H\left(S\right)-\sum_{i=1}^{n}H\left(X_{i}\right)-H\left(S\right)=H\left(S\right)-H\left(S\right). \end{array}$$

The above expression is always non-negative because the distribution of S has the maximum entropy in a set where the distribution of *S* belongs. Seeing that the entropy quantifies the average uncertainty of a random variable, H(S)−H(S) represents the information given by the existing correlations in *S*, that is not incorporated in the structural relations from S.

## 3. Proposed Method

We now set out to derive the proposed EC method for temporal data. For convenience, we start by introducing a few additional notations. Let a dataset *D* be a collection of pairs $\left(T_{j},c_{j}\right)$ for all $j\in \left\{1,\ldots ,N\right\}$, where $T_{j}$ consists of a TS, $c_{j}$ corresponds to its respective class label and *N* is the number of instances in *D*. In general, a TS is defined as a vector of length *L*

(8) $$T_{j}=\left(x_{1}^{\left(j\right)},x_{2}^{\left(j\right)},\ldots ,x_{L}^{\left(j\right)}\right),$$

where each component $x_{k}^{\left(j\right)}=\left(x_{k1}^{\left(j\right)},x_{k2}^{\left(j\right)},\ldots ,x_{km}^{\left(j\right)}\right)$ consists of *m* features measured at *time point* (TP) k∈{1,…,L}. The object of TS classification is to associate a class label *c* to a given time-series *T* (not necessarily in the data).

Consider a TS *T*, as in Equation (8), representing the evolution of the random vector $X=(X_{1},\ldots X_{m})$ over time, and its respective class label, which is denoted by the random variable *C*. The set of $X_{k}$ can be viewed as a collection of time-dependent discrete random variables, for which a joint probability distribution can be defined. Note that, since a TS is chronologically organized, it is relevant to analyze the dependence of variables on their early states, i.e., the degree of dependence of X at a certain TP on the value observed at a previous instant. Similarly, the correlation between *C* and $X_{k}$ quantifies the dependence that the vector X at TP *k* has on the class label. In the EC context, the focus is to study systems where the class labels verify a high dependence on a certain amount of early states of $X_{k}$, while the remaining TPs are dispensable for a satisfactory classification.

Consider the finite set of discrete random variables *S* to be composed of the TS *T* together with its respective class label *C*. The system

$$S=\left\{X_{1},X_{2},\ldots ,X_{n},X_{n+1},\ldots ,X_{L},C\right\}$$

has an associated joint probability distribution $P_{S}\left(X_{1},X_{2},\ldots ,X_{L},C\right)$, where *L* represents the TS length. The goal is to find the value *n* and the distribution $P_{S}^{n}$, such that $P_{S}^{n}\left(C|X_{1},X_{2},\ldots ,X_{n}\right)\approx P_{S}\left(C|X_{1},X_{2},\ldots ,X_{L}\right)$. Therefore, $P_{S}^{n}\left(C|X_{1},X_{2},\ldots ,X_{n}\right)$ and $P_{S}\left(C|X_{1},X_{2},\ldots ,X_{L}\right)$ describe the probability of the class label *C* occurring, provided that the first *n*, or all variables of *T*, are known, respectively.

In some cases, the joint probability distribution $P_{S}$ is not known in advance; thus, it has to be computed from the data, through maximum likelihood estimation. In particular, for a dataset *D* of size *N*, the distribution $P_{S}$ that maximizes the likelihood of *D* is such that

(9) $${\hat{P}}_{S}\left(X_{1}=x_{1},\ldots X_{L}=x_{L},C=c\right)=\frac{|D_{x_{1},\ldots ,x_{L},c}|}{N},$$

where $|D_{x_{1},\ldots ,x_{L},c}|$ is the number of instances in *D* for which $X_{i}$ takes the value $x_{i}$ and *C* the value *c*.

**Figure 1:**
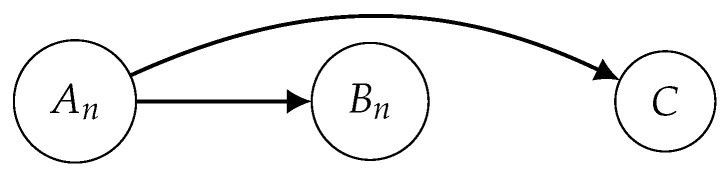
Bayesian network representation of the structure $S_{n}$ from the system *S*.

Given the system *S*, the set of structural relations, defined by

$$S_{n}=\left\{\left\{X_{1},\ldots ,X_{n},X_{n+1},\ldots ,X_{L}\right\},\left\{X_{1},\ldots ,X_{n},C\right\}\right\},$$

depends on the value of *n* and it corresponds to a structure that respects the previously described properties. Considering the sets $A_{n}=\left\{X_{1},\ldots ,X_{n}\right\}$ and $B_{n}=\left\{X_{n+1},\ldots ,X_{L}\right\}$, the structure is represented as $S_{n}=\left\{\left\{A_{n},B_{n}\right\},\left\{A_{n},C\right\}\right\}$. The structural relation $A_{n}$ contains information about the evolution of the variable X until the TP *n*, i.e., the early stages of the collection of TS. On the other hand, $B_{n}$ describes the remaining instants of $T_{i}$, which can be viewed as the knowledge about the later stages of the variable X. Finally, *C* represents the class label information from the collection of TS. The structure $S_{n}$ can be seen as a simplified model of the system *S*. It is expected to include the correlations between the early and the later information about the TS ($A_{n}$ and $B_{n}$), as well as between the early states of $T_{i}$ and the knowledge about their classes ($A_{n}$ and *C*). Conversely, the correlations between $B_{n}$ and *C* are not preserved because the idea is to study the possibility of describing the class from the early states $A_{n}$, while neglecting the information from $B_{n}$. The probability distribution of $S_{n}$ is obtained based on Theorem 1 (the proof can be found in Appendix A) and considering the BN represented in Figure 1.

**Theorem** **1.** *Consider the Bayesian network*$B_{n}=\left(G_{n},\Theta_{n}\right)$*with*$G_{n}$*given by Figure 1 and*$\Theta_{n}$*calculated according to Equation* (2)*. The structure* $\left(S_{n},P_{S_{n}}\right)$ *over S has a probability distribution equal to the joint probability distribution of* $B_{n}$*, that is,* $P_{S_{n}}=P_{B_{n}}$*.*

Given the structure of the Bayesian network from Figure 1, we have $\Pi_{A_{n}}=\emptyset$, $\Pi_{B_{n}}=\left\{A_{n}\right\}$ and $\Pi_{C}=\left\{A_{n}\right\}$, and by Equation (1) we have:

(10) $$P_{S_{n}}=P\left(A_{n}\right)P\left(B_{n}|A_{n}\right)P\left(C|A_{n}\right).$$

where each (conditional) probability is obtained by the observed frequencies given by Equation (2). From Equation (7) and for each value of *n*, the difference of entropy applied to these context is represented as:

(11) $$I\left(S\right)-I\left(S_{n}\right)=H\left(S_{n}\right)-H\left(S\right).$$

As stated above, we want to measure the loss of information about class when we lose correlations. This can be performed using conditional entropy, namely

(12) $$H_{P_{S_{n}}}\left(C|X_{1}\ldots X_{L}\right)-H_{P_{S}}\left(C|X_{1}\ldots X_{L}\right)=H\left(C|A_{n}\right)-H\left(C|A,B\right),$$

where $A,B=A_{n},B_{n}$ (note that $A_{n},B_{n}=X_{1}\ldots X_{L}$ for all *n*). The conditional entropy is used to quantify the uncertainty about the classes of the collection of TS, given that *T* is fully or partially known. On the one hand, $H(C|A_{n})$ consists of the amount of information required to predict the class labels, provided that the TS are known until the TP *n*. On the other hand, H(C∣A,B) corresponds to the amount of information needed to describe *C*, based on the knowledge of the entire *T*. The difference between these two conditional entropies measures the knowledge that the whole TS provides about the classes (correlation between *C* and A,B), which is not represented by the incomplete data (correlation between *C* and $A_{n}$). Thus, Equation (11) can be viewed as the lack of information caused by describing the structural relation *C* from $A_{n}$, i.e., the loss of knowledge for using the collection of TS only until the early TP in the classification process.

In addition to earliness in predicting the classes, the goal consists of finding the value *n* for which $S_{n}$ represents the system *S* with reasonable complexity. Since this can be seen as a problem of learning the BN from Figure 1, both MDL and AIC scores are applied to the multivariate correlations for EC approach, in the interest of finding the best fitting model. These scores are used as two criteria for choosing the early TP, such that the selection of the model takes its simplicity into consideration. From Equation (3) and considering $P_{S_{n}}$, described in Equation (10), the MDL score is defined as

(13) $$\begin{array}{ccc} MDL(D|S_{n}) & = & \frac{\log_{2}N}{2}|S_{n}|-\sum_{i=1}^{N}\log_{2}\left[P_{S_{n}}\left(A_{n},B_{n},C\right)\right]\\ & = & \frac{\log_{2}N}{2}|S_{n}|-\sum_{i=1}^{N}\log_{2}\left[P\left(C|A_{n}\right)P\left(B_{n}|A_{n}\right)P\left(A_{n}\right)\right], \end{array}$$

where *N* is the number of instances in the dataset *D*, $|S_{n}|$ denotes the number of independent parameters in the model, and $P_{S_{n}}$ is the underlying distribution associated to the structure $S_{n}$, which describes *S* as a representation of the given data. Similarly, the AIC score, applied to this context, is defined as:

(14) $$\begin{array}{ccc} AIC(D|S_{n}) & = & |S_{n}|-\sum_{i=1}^{N}\log_{2}\left[P_{S_{n}}\left(A_{n},B_{n},C\right)\right]\\ & = & |S_{n}|-\sum_{i=1}^{N}\log_{2}\left[P\left(C|A_{n}\right)P\left(B_{n}|A_{n}\right)P\left(A_{n}\right)\right]. \end{array}$$

As represented in the direct acyclic graph structure from Figure 1, the goal is to analyze how the structural relation $A_{n}$ is able to describe *C*, while the correlation between $B_{n}$ and *C* is neglected. For this reason, the computation of the network complexity only considers the relation between the early states and the class labels,

(15) $$|S_{n}\left|=\right|\left\{A_{n},C\right\}\left|=|\right|A_{n}\left|\right|-1+\left(||C||-1\right)\left|\right|A_{n}\left|\right|=\left|\right|A_{n}\left|\right|\times \left||C|\right|-1,$$

where $\left|\right|A_{n}\left|\right|$ and ||C|| denote the number of distinct observations in the structural relation $A_{n}$ and *C*, respectively. In Equations (13) and (14), the first term quantifies the complexity of the model, i.e., the amount of information required to encode not only $S_{n}$, but also the data given $S_{n}$. The second term measures the LL of the data based on the model, i.e., the amount of information needed to represent the dataset *D* according to the probability distribution $P_{S_{n}}$. As *n* increases, the size of $A_{n}$ becomes larger, the number of correlations is higher and, consequently, the complexity of the model increases. In addition, the more information about the TS there is, the better the correlations describe the data, which means a decrease in the number of bits needed to describe *C* from $A_{n}$. The difference between these two terms describes the trade-off between the model complexity and the effectiveness of the fit to the data. The simplest model, that is able to use the least amount of correlations while maintaining a distribution as close to the original as possible, is found through minimizing both $MDL(D|S_{n})$ and $AIC(D|S_{n})$.

The Multivariate Correlations for Early Classification (MCEC) procedure, summarized in Algorithm 1, receives as input a comma-separated values (CSV) file, containing the TS and the respective class labels, and a scoring function ϕ. Both univariate and multivariate TS are allowed; however, the TS must be of fixed length. For both AIC and MDL, the overall time complexity of the MCEC algorithm is $O(L^{3}N^{2}\log^{2}\left(N\right))$, where *L* is the size of the time-series and *N* is the number of time-series in the data. Indeed, the procedure needs to store and count at most *N* configurations of $A_{n}$, and checking whether a configuration of $A_{n}$ (with size O(L)) already occurred takes O(Llog(N)) time (using a binary search tree such as an AVL tree). Moreover, for each configuration of $A_{n}$ the procedure needs to store and count at most *N* configurations of $B_{n}$ (to establish the frequencies $P(B_{n}|A_{n})$), which leads to an overall time complexity of $O(L^{2}N^{2}\log^{2}\left(N\right))$ for Step 3.
**Algorithm 1** MCEC algorithm1: **for**
$n\in \left\{1,\ldots ,L\right\}$
**do** 2:    Compute $S_{n}=\left\{\left\{A_{n},B_{n}\right\},\left\{A_{n},C\right\}\right\}$ 3:    Compute $\phi (D|S_{n})$ 4:    If $\phi (D|S_{n})$ is minimum, store *n* 5: Output stored *n*

## 4. Experimental Results

The proposed algorithm was implemented in Java language, and is freely available in GitHub at https://joaopbeirao.github.io/MCECalgorithm/. In the interest of verifying the reliability of the proposed EC approach, an investigation on the accuracy of multiple classifiers was done. In this regard, we vary the length of the TS and check if the proposed cut-points attain a similar accuracy when compared with the complete TS. We note that the purpose of this assessment is not to elicit the best classifier among those used, but rather to understand the consequences of using a truncated time-series in classification tasks. Seven classifiers were considered, using the default parameters and stratified cross-validation with 10 folds: Naïve Bayes (NB), Bayes Net (BN), Sequential Minimal Optimization (SMO), C4.5 decision tree (J48), Reduces Error Pruning Tree (REPTree), Forest of Multiple Random Trees (RandomForest) and *k*-Nearest-Neighbor (*k*NN). We performed classification using the classifiers from the Weka Data Mining Software (version number 3.8) [25]. All the experiments were conducted using a PC with an Intel Core i7-2677M@1.80 GHz CPU and with 4 GB RAM.

From the MCEC algorithm, for each dataset, three values for the EC TP (*n*) were extracted. The first value is obtained from the difference in entropy measure: the smallest *n*, such that $H\left(C|A_{n}\right)-H\left(C|A,B\right)\leq 0.3\times \left[H\left(C|A_{1}\right)-H\left(C|A,B\right)\right]$, which means that *n* corresponds to the TP where a reduction of 70% from the initial value of entropy is verified, henceforth called CH−70, defined as the score:

$$CH-70\left(D|S_{n}\right)=\left\{\begin{array}{cc} +\infty & \mathrm{if}H\left(C|A_{n}\right)-H\left(C|A,B\right)>0.7\left(H\left(C|A_{1}\right)-H\left(C|A,B\right)\right)\\ n & \mathrm{otherwise}. \end{array}\right.$$

The second and third values are the result of minimizing $MDL(D|S_{n})$ and $AIC(D|S_{n})$, respectively, i.e., *n* consists of the TP where the scores are minimum.

For analyzing the performance of the MCEC algorithm, we use thirteen benchmark datasets from the UEA & UCR Time Series Classification Repository [26,27]. This subset of examples is considered representative, as it comprises a diverse range of both dimensional parameters and classification conditions. Each dataset is composed of numeric TS with a fixed length, and their respective class labels.

For each example, a training set and a test set are provided separately. The preprocessing of the data included the aggregation of both training and test subsets in one single dataset. In addition, a TS discretization was performed following the guidelines proposed by Lin et al. [28] and Hu et al. [29]; no feature selection was performed. None of the datasets contained missing values; therefore, no imputation was required. The description of the data used in the experiments is given in Table 1. For each dataset, the number of class values (#*C*), the number of variables for each TP (*m*), the length of the discretized TS (*L*), the number of instances (*N*), and the type of data are provided.

The results from Table 2 describe the MCEC algorithm effort in attempting EC, based on the analysis of the information contained in the datasets. For each dataset, it is given the Earliness as the percentage value associated with the EC TP *n* computed from MCEC algorithm: $\mathrm{Earliness}\left[\%\right]=\frac{n}{L}\times 100.$ The Accuracy columns present only the best (10-fold cross-validation) classification result (among all classifiers used) for the given data, predicting the class variable at TP *n*. The column Full contains the best outcome (among all classifiers used) for the complete TS, and it is used as a reference framework.

The results from the Earliness column of Table 2 confirm that in most datasets, it is viable to perform early classification. Concerning the CH−70 and AIC, the classification accuracy with fewer TPs outperforms the reference value (Full column) for the Car and ECG200 dataset. These examples suggest that it is possible to obtain a better classification performance using only part of the TS (in these cases, around 50% or even less) from the data. In the case of the Computers dataset, AIC indicates the use of 80% of the TS, attaining with it an accuracy very close to the reference value. A similar result is obtained with the Meat dataset, where AIC pointed out to use just over half the size of the TS. In the multivariate ECG200M dataset, AIC pinpoint the use of only 15% of the MTS obtaining a difference of 1% less in accuracy, when compared with the reference value in the Full column. In other datasets (ArrowHead, BirdChicken, SynthControl, Wafer, and WaferM) accuracy outcomes with at most −3% in comparison with the full-length result are obtained. This means that, in these experiments, with fewer TPs analyzed (earlier in time), the loss in terms of classification accuracy can be diminished.

Regarding the scoring metrics, MDL always proposes the lowest values for the early classification TP (Earliness column). However, the corresponding classification accuracy results of CH−70 and AIC outperform the ones for MDL in all cases. Moreover, CH−70 achieves higher classification accuracy in six out of thirteen cases, AIC in three datasets, and in the remaining four instances, a draw is verified between the CH−70 and AIC. Therefore, from the experimental tests described in Table 2, in general, CH−70 achieves better results, in terms of classification accuracy, and MDL demonstrates a superior earliness ability. AIC evidences the foremost competence in balancing these two targets. Nevertheless, the early classification capabilities of the MCEC algorithm are acknowledged, seeing that this context is based on the trade-off between these two main objectives: accuracy and earliness.

### 4.1. Analysis of the ECG200 and Car Results

Herein, we detail the analysis of two datasets that illustrate the merits of the proposed method, complementing Table 2 with a graphical interpretation of the results, drawing conclusions both in terms of the relevant fraction of the time-series data for classification and the quality of the proposed scoring criteria.

**Figure 2:**
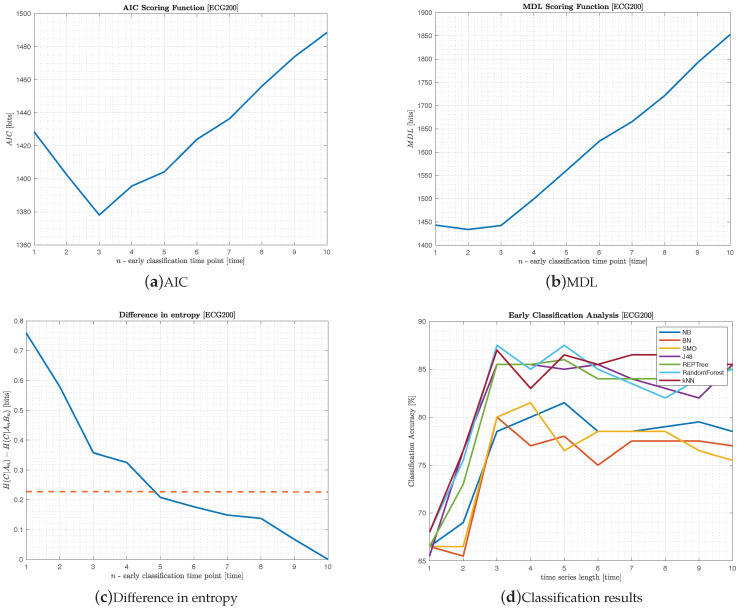
Experimental results of the MCEC algorithm on the ECG200 dataset.

The ECG200 data comprises two hundred time-series that traces the electrical activity recorded during one heartbeat used as part of R. Olszewski Ph.D. thesis [30]. It aims to discriminate between normal heartbeat and myocardial infarction. The original time-series has 96 TPs. After discretization, following the guidelines proposed by Hu et al. [29], the time-series was reduced to ten TPs. The results are depicted in Figure 2.

Results suggest that only a fraction of the heartbeat signal is crucial to discriminate between classes. In particular, AIC (Figure 2a) and CH−70 (Figure 2c) proposed to cut the time-series at TPs 3 and 5, respectively; in CH−70 the cut point corresponds to the first *n* bellow the dashed red line. Indeed, both cut points proposed by AIC and CH−70 agree upon a peak in accuracy of 87.5% (c.f. Table 2), and so surpassing the full-length time-series in 2%. The MDL criterion is more confident in this regard (c.f. Figure 2b), proposing to cut the time-series at n=2. However, in this particular case, it suggested a premature cut leading to lower prediction values, as depicted in Figure 2d.

**Figure 3:**
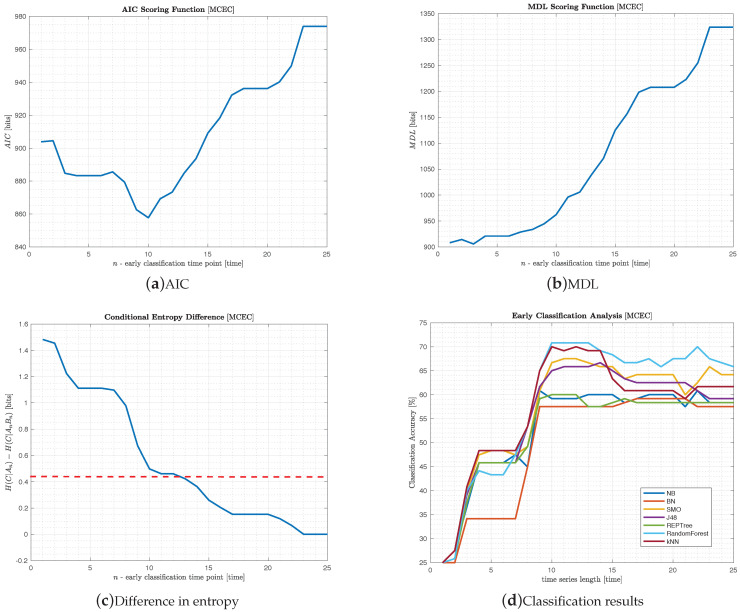
Experimental results of the MCEC algorithm on the Car dataset.

In addition, the experimental results on the Car example are represented in Figure 3. Figure 3a,b represent the variation of $AIC(D|S_{n})$ and $MDL(D|S_{n})$, respectively, for $n\in \left\{1,\ldots ,25\right\}$. While for AIC, the minimum is reached at n=10 (corresponding to 40% of 25 TPs), for MDL, the lowest value is attained at n=3 (corresponding to 12% of 25 TPs). In both cases, this extreme is followed by an irregular growth until n=23, where it stabilizes at a maximum value. Figure 3c describes the behaviour of $H\left(C|A_{n}\right)-H\left(C|A,B\right)$ while varying *n* from 1 to L=25. A decrease of 70% from the initial entropy value is obtained at n=13 (corresponding to 52% of 25 TPs), depicted with a dashed red line.

Figure 3d includes the classification accuracy of the Car dataset. Note that there are two jumps in classification accuracy at n=3 and n=10, corresponding precisely to the timestamps elicited by the MDL and AIC, respectively.

### 4.2. Statistical Significance

Experimental results were compared with statistical significance tests in order to understand the benefit of the trade-off between the two main goals in EC: accuracy and earliness. Among the tested datasets, the MCEC algorithm provided a value of *n*, with an associated percentage (Earliness). For each situation, the group of classifiers determined the Accuracy value. In addition, the classification of the full-data worked as a reference framework: no earliness and complete TS accuracy. In order to represent the balance between these two requirements, we consider the following quantity BEA(p)=p×(100−E)+(1−p)×A, where *E* and *A* correspond to the Earliness and Accuracy percentages, respectively, and *p* consists of the weight that determines the relevance given to each variable. Seeing that an accurate classification is desirable, as early as possible, BEA describes the management of the two fundamental challenges of the EC problem. The thirteen datasets from Table 2 were considered, as well as their respective values of *E* and *A*, for each of the three measures that compose the MCEC algorithm, together with the reference framework. Note that all Full outcomes verify E=0%, since the entire collection of TS is considered for classification.

Table 3 includes the results of the Wilcoxon signed-rank sum test [31], for comparing the performance of classification using the MCEC algorithm timestamp *n* on the TS. These tests examine the relation between the scores in pairs, in order to verify if there is enough evidence to claim that the differences are significant, for a significance level of α=0.05. The arrow in Table 3 points towards the measure with better performance, according to the value of $p\in \left\{0,0.25,0.5,0.75\right\}$. The double arrow means there is enough evidence to claim the difference is significant.

The results demonstrate that, for p=0, there is enough evidence to claim that Full surpasses all other measures. Furthermore, between CH−70 and the other two model selection criteria, the difference in entropy outperforms both scoring functions, and AIC shows better results than MDL. All these differences are statistically significant. For p=0.25, CH−70 has the best performance in comparison with all of the remaining. The AIC measure seems to achieve significantly superior results than MDL; however, there is not enough evidence to claim that AIC outperforms Full, nor that the latter surpasses MDL. For p=0.5, the only assurance consists of Full performing the worst. Among CH−70, MDL, and AIC, the differences between them are not statistically significant. Lastly, at p=0.75, Full continues to be surpassed by all the others, as well as the difference in entropy in comparison with both model selection criteria. However, between MDL and AIC, there is not enough evidence to confirm which performs the best.

## 5. Conclusions

This work proposes a novel algorithm, named MCEC, that aids in addressing the challenges associated with the task of early prediction in (univariate and multivariate) time-series data. Existing methods yield, for each time-series sample, a timestamp from which it is possible to perform early classification, failing to provide an overall data perspective. To the best of our knowledge, MCEC is the first approach that can grant the early opportunity of the entire data, allowing us to reason about prediction outcomes after understanding data idiosyncrasies.

MCEC is very flexible as it can be used with different scoring criteria, allowing for the trade-off between earliness and accuracy. We propose three measures: CH−70, MDL and AIC. The achieved results confirm the ability of the MCEC method to examine the EC opportunity within a dataset. In general, the three criteria are capable of choosing a timestamp for which the time-series classification is plausible. Overall, the CH−70 obtains better accuracy results, MDL demonstrates a superior tendency for earliness, and AIC attains the most competent balance between both aims. Examples, where the earliness is very low, may indicate that, given the information available, the criterion recognized that the increase in the knowledge obtained from the data did not justify the growth in the model complexity required for its description. Conversely, the AIC results demonstrate a more adventurous disposition in choosing the value for the early classification timestamp, which produced a relative success in benchmark data.

In terms of future work, several machine learning tasks can be developed based on the capabilities of this information-theoretic approach. A classification method can take profit from it, giving different attention to time-series whose classification timestamp deviates from that derived from the data; for instance, inspecting only part of electrocardiograph signals might improve existing classification methods [32]. It can also aid in determining the change point detection, as well as feature extraction and selection methods from multivariate time-series data. In the latter case, for instance, a greedy feature selection could be performed based not only on the difference in entropy measure but also on the model selection criteria. Finally, MCEC, if applied in a time-series from back/present to front/past, has the potential of unraveling the optimal Markov length of the stochastic process underlying the data.

## Figures and Tables

**Table 1:** Description of the benchmark time-series used in the experiments.

Dataset	#*C*	*m*	*L*	*N*	Type
ArrowHead	3	1	14	211	image
BirdChicken	2	1	25	40	image
Car	4	1	25	120	sensor
CBF	3	1	12	930	simulated
Coffee	2	1	71	56	spectro
Computers	2	1	5	500	device
ECG200	2	1	10	200	EGC
GunPoint	2	1	150	200	motion
Meat	3	1	7	120	spectro
SynthControl	6	1	6	8	simulated
Wafer	2	1	152	7164	sensor
ECG200M	2	2	13	200	ECG
WaferM	2	6	13	1195	sensor

**Table 2:** Experimental results of the MCEC algorithm.

Dataset	Earliness			Accuracy			Full
	CH−70	MDL	AIC	CH−70	MDL	AIC	
ArrowHead	57.14%	7.14%	42.86%	72.51%	38.39%	70.14%	78.20%
BirdChicken	20.00%	4.00%	8.00%	62.50%	57.50%	75.00%	77.50%
Car	52.00%	12.00%	40.00%	70.83%	40.83%	70.83%	65.83%
CBF	66.67%	33.33%	66.67%	91.61%	74.09%	91.61%	94.95%
Coffee	19.72%	9.86%	9.86%	85.71%	76.79%	76.79%	98.21%
Computers	20%	20%	80%	51.20%	51.20%	60.80%	61.40%
ECG200	50.00%	20.00%	30.00%	87.50%	76.50%	87.50%	85.50%
GunPoint	24.00%	0.67%	15.33%	92.00%	71.50%	83.00%	99.50%
Meat	28.57%	28.57%	57.14%	69.00%	69.00%	74.50%	75.50%
SynthControl	62.50%	25.00%	50.00%	89.50%	62.67%	85.00%	92.33%
Wafer	7.24%	1.32%	1.97%	97.91%	97.60%	97.66%	99.85%
ECG200M	38.46%	7.69%	15.38%	80.50%	73.00%	80.00%	81.00%
WaferM	30.77%	7.69%	30.77%	93.22%	89.36%	93.22%	96.15%

**Table 3:** Comparison of the classification using the three MCEC algorithm cuts (CH−70, AIC and MDL) and Full TS against each other, using the Wilcoxon signed-rank sum test applied to the trade-off experimental data, scored according to BEA(p).

Comparison		*p*			
		0	0.25	0.5	0.75
CH−70⇔MDL	size	26	26	26	26
	*p*-value	<0.01	<0.01	0.81	<0.01
	better	⇐	⇐	→	⇒
CH−70⇔AIC	size	25	26	26	26
	*p*-value	<0.01	0.01	0.34	<0.01
	better	⇐	⇐	→	⇒
CH−70⇔ Full	size	26	26	26	26
	*p*-value	<0.01	<0.01	<0.01	<0.01
	better	⇒	⇐	⇐	⇐
MDL⇔AIC	size	20	20	20	20
	*p*-value	<0.01	<0.01	0.23	0.37
	better	⇒	⇒	→	←
MDL⇔ Full	size	26	26	26	26
	*p*-value	<0.01	0.70	<0.01	<0.01
	better	⇒	→	⇐	⇐
AIC⇔ Full	size	26	26	26	26
	*p*-value	<0.01	0.37	<0.01	<0.01
	better	⇒	←	⇐	⇐

**Figure A1:**
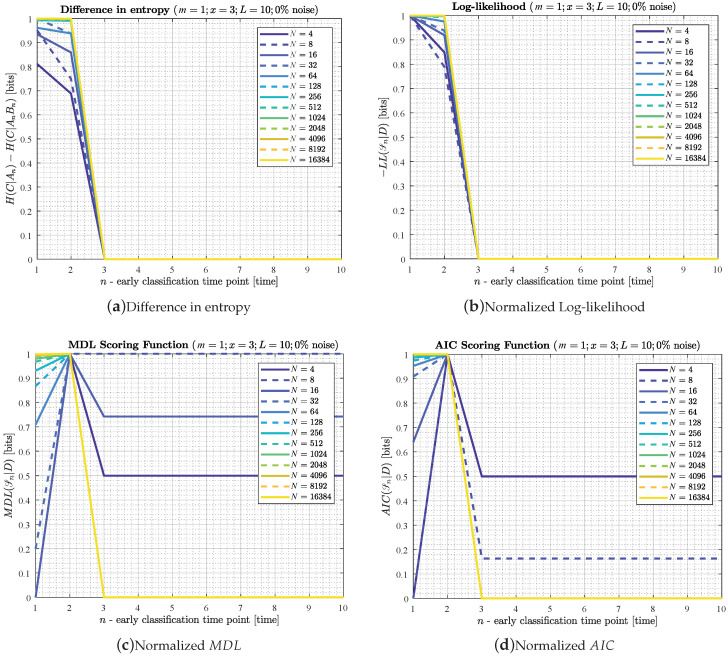
Variation of *n* for synthetic datasets with m=1, L=10, x=3, pNoise=0%.

**Figure A2:**
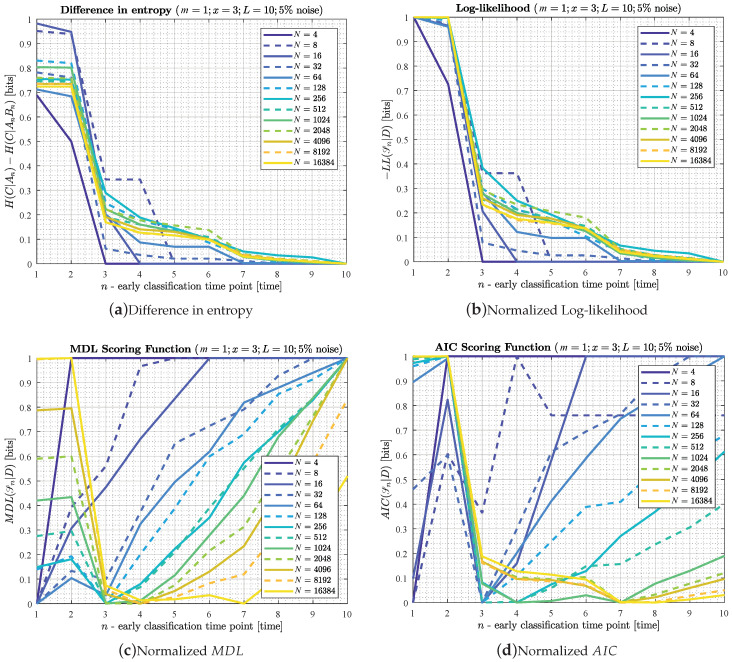
Variation of *n* for synthetic datasets with m=1, L=10, x=3, pNoise=5%.

**Figure A3:**
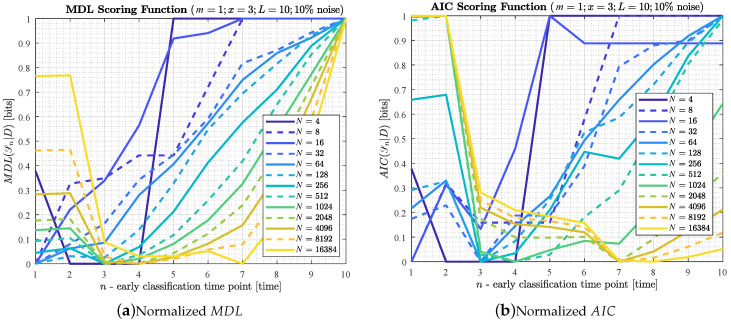
Variation of *n* for synthetic datasets with m=1, L=10, x=3, pNoise=10%.

**Figure A4:**
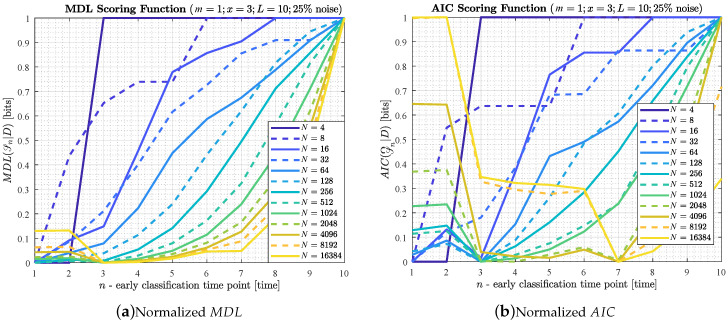
Variation of *n* for synthetic datasets with m=1, L=10, x=3, pNoise=25% and stuff.

## Appendix A. Proof of Theorem 1

The proof is based on the Lagrange multiplier method. For the sake of simplicity, take $A=A_{n}$ and $B=B_{n}$. Denote

$$p_{abc}={\hat{P}}_{S}\left(A=a,B=b,C=c\right)$$

and

$$q_{abc}={\hat{P}}_{S}\left(B=b|A=a\right){\hat{P}}_{S}\left(C=c|A=a\right){\hat{P}}_{S}\left(A=a\right)=P_{B_{n}}\left(A=a,B=b,C=c\right).$$

We proceed to show that $q_{abc}$ is a stationary point of the entropy function

$$H\left(q_{abc}\right)=-\sum_{a,b,c}q_{abc}\log\left(q_{abc}\right)$$

under the constrains

- $p_{ab}=\sum_{c}p_{abc}=\sum_{c}q_{abc}=q_{ab}$,
- $p_{ac}=\sum_{b}p_{abc}=\sum_{b}q_{abc}=q_{ac}$, and
- $\sum_{a,b,c}q_{abc}=1$.

By the concavity of entropy, any solution to the previous problem is a maximum. Using the Lagrange multiplier method, we look for a maximum of the function

$$L\left(q_{abc}^{\prime }\right)=H\left(q_{abc}^{\prime }\right)-\sum_{a,b}\lambda_{ab}\left(q_{ab}^{\prime }-p_{ab}\right)-\sum_{a,c}\lambda_{ac}\left(q_{ac}^{\prime }-p_{ac}\right)-\lambda_{u}\left(\sum_{a,b,c}q_{abc}^{\prime }-1\right),$$

where the $\lambda_{ab},\lambda_{ac}$ and $\lambda_{u}$ are Lagrange multipliers. It is now enough to show that by setting $q_{abc}^{\prime }=q_{abc}$, and for adequate values of the Lagrange multipliers, $\frac{\partial L}{\partial q_{abc}^{\prime }},\frac{\partial L}{\partial \lambda_{ab}},\frac{\partial L}{\partial \lambda_{ac}},\frac{\partial L}{\partial \lambda_{u}}$ all vanish. Effectively,

$$\begin{array}{cc} {\left.\frac{\partial L}{\partial q_{abc}^{\prime }}\right|}_{q_{abc}} & =-{\left.(\log\left(q_{abc}^{\prime }\right)+1+\lambda_{ab}+\lambda_{ac}+\lambda_{u})\right|}_{q_{abc}}\\ \; & =-(\log\left(p_{ab}p_{ac}/p_{a}\right)+1+\lambda_{ab}+\lambda_{ac}+\lambda_{u})\\ \; & =\log\left(p_{a}\right)-\log\left(p_{ab}\right)-\log\left(p_{ac}\right)-1-\lambda_{ab}-\lambda_{ac}-\lambda_{u} \end{array}$$

vanishes by setting

$$\begin{array}{c} \lambda_{ab}=\frac{1}{2}\log\left(p_{a}\right)-\log\left(p_{ab}\right),\;\;\;\;\lambda_{ac}=\frac{1}{2}\log\left(p_{a}\right)-\log\left(p_{ac}\right),\;\;\;\;\lambda_{u}=-1. \end{array}$$

On the other hand, we have that

$$\begin{array}{c} {\left.\frac{\partial L}{\partial \lambda_{ab}}\right|}_{q_{abc}}={\left.(q_{ab}^{\prime }-p_{ab})\right|}_{q_{abc}}=\sum_{c}q_{abc}-p_{ab}=\sum_{c}\frac{p_{ab}p_{ac}}{p_{a}}-p_{ab}=p_{ab}-p_{ab}=0\\ {\left.\frac{\partial L}{\partial \lambda_{ac}}\right|}_{q_{abc}}={\left.(q_{ac}^{\prime }-p_{ac})\right|}_{q_{abc}}=\sum_{b}q_{abc}-p_{ac}=\sum_{b}\frac{p_{ab}p_{ac}}{p_{a}}-p_{ac}=p_{ac}-p_{ac}=0. \end{array}$$

Finally,

$$\begin{array}{c} {\left.\frac{\partial L}{\partial \lambda_{u}}\right|}_{q_{abc}}={\left.\sum_{a,b,c}q_{abc}^{\prime }-1\right|}_{q_{abc}}=\sum_{a,b,c}q_{abc}-1=0, \end{array}$$

as $q_{abc}$ is a probability distribution by construction. Therefore, $q_{abc}=P_{B_{n}}$ satisfies the definition of the probability distribution associated with the structure $S_{n}=\left\{\left\{A,B\right\}\left\{A,C\right\}\right\}$, and therefore, it matches $P_{S_{n}}$.

## Appendix B. Results on Synthetic Data

Herein we describe the empirical study of the proposed method on synthetically generated datasets. The procedure to generate synthetic data is based on the exclusive disjunction, and it allows an interpretation of the results in comparison with the expected outcomes. The parametrization of the data generator enables the variation on standard time-series dataset aspects: the number of features per time point (*m*), the length of the time-series (*L*), and the number of instances (*N*). Moreover, two additional variables are included: the number of randomly generated columns (*x*) and the percentage of noise in the dataset (pNoise). Recall the data type is boolean for all features, and all datasets contain 2 classes. According to the specified parameters, a database is created, with *N* time-series, each with *m* attributes per time point and length equal to *L*. The value of *x* represents the number of initial instants that are randomly generated. The following time points are computed as the XOR of the *x* previous ones. For the multivariate case, where each instant is composed of a set of features, the process is maintained for each attribute independently. The class labels are computed with the same use of the exclusive disjunction, however, for m≥2, another XOR is applied to the collection of features, in order to obtain only one value for the class attribute. Aiming for providing more realistic data, the noise is used, causing a number of arbitrary positions to be switched, i.e., 0 becomes 1 and vice versa.

The idea is to produce a set of time-series where the class labels are a function of the *x* initial time points in the interest of analyzing if the proposed algorithm can recognize this correlation and, consequently, the early classification opportunity.

At the first stage, the impact of the dimensional parameter variation on the MCEC method behavior is analyzed. Seeing that the two model selection criteria used in the proposed approach are sensitive to the data size, the variation of the number of variables *m* is studied under different conditions. Therefore, the output graphs are examined except for the classification accuracy, since the intention is to explore how the system is affected by modifications in the size of the dataset. The absolute values of the log-likelihood and of both scoring functions increase with the number of variables *m*. Because of that, feature scaling normalization, given by:

(A1) $$x^{\prime }=\frac{x-x_{min}}{x_{max}-x_{min}},$$

is applied to the results, for comparing the relative behaviour of the quantities. Only the entropy graph includes the non-normalized values, on account of being a difference between two variables of the same order of magnitude.

Figure A1 represents the behavior of the four measures under investigation for datasets with a different number of instances.

As previously mentioned, $H\left(C|A_{n}\right)-H\left(C|A,B\right)$ quantifies the lack of knowledge caused by describing the classes using the time-series in the dataset only until time point *n*. From Figure A1a, the variation of *m* does not extensively affect the difference in entropy. Since $H\left(C|A_{n}\right)-H\left(C|A,B\right)=0$ for n≥3, there seems to be enough information to predict the class labels, with the first three-time points. The variation of entropy from n=1 to n=2 is sharper for lower values of *N*, and it becomes null while the number of instances increases.

Furthermore, $-LL(D|S_{n})$ describes the amount of information needed to represent the dataset *D* using the model $S_{n}$. Figure A1b demonstrates that the data is entirely depicted by the structure $S_{3}$, seeing that the normalized log-likelihood is zero from n=3 forward. The behavior of this measure is very similar to the difference in entropy since they both quantify how good the model fits the data.

Regarding the scoring functions, in the early classification context, the lowest value of both scores corresponds to the time point from which additional information can be disregarded. While the graph from Figure A1c shows that MDL has a minimum at n≥3 for N≥32, the one from Figure A1d displays AIC achieving it for N≥16. In the two cases, the scores are constant from the point where they attain the lowest value. This is because each time point is a function of what is behind since it consists of the exclusive disjunction of the three previous instants. Consequently, the number of independent parameters in group $A_{n}$ ($\left|\right|A_{n}\left|\right|$) is constant for n≥3, i.e., the number of distinct cases in the list $A_{n}$ stabilises from that point on.

Three columns are randomly generated (x=3), which means that a feasible prediction of the class labels is expected using only the first three-time points. This figure describes the univariate case (m=1), for a fixed time-series length (L=10), with no addition of noise (pNoise=0%). In order to explore a dimensional range for the data size, the values for *m* comprise a set of powers of two: $\left\{2^{2},2^{3},\ldots ,2^{14}\right\}$.

Figure A2 describes the experimental tests in datasets with the same parameters as the ones used in Figure A1, except for the percentage of noise.

With pNoise=5%, a more realistic environment is simulated. The difference in entropy (Figure A2a) and the log-likelihood (Figure A2b) have a smoother decreasing behavior and more difficulty in reaching zero, in particular for higher values of *m*. However, in general, the most significant reduction in both cases is verified from n=2 to n=3. This indicates that, although the lack of information is minimized as more of the time-series is observed, for a certain threshold, the graphs show an early classification opportunity. Similarly to the 0% noise case, the increase in the number of instances is followed by a stabilization from n=1 to n=2, which means that less knowledge is gained with the use of only the first two time points. The behavior of both measures seems to be convergent for N→∞.

Regarding the scoring functions, Figure A2c and Figure A2d show a high variance of MDL and AIC with the data size, respectively. For few instances, the lowest value is obtained at n=1, which means that the model, considered the best in terms of complexity and fitness to the data, is the one with merely the first time point. In this case, a proper model selection is impracticable, since the samples available are insufficient to extract the model conveniently; that is, the data does not contain enough information. MDL displays a minimum at n≥3 for N≥128 (higher than for 0% noise) and AIC for N≥16 (the same as for 0% noise). The lowest value of $MDL(D|S_{n})$ is attained at n=3 for $N\in \left\{128,\ldots ,1024\right\}$, at n=4 for $N\in \left\{2048,\ldots ,8192\right\}$, and at n=7 for N=16,384. The minimum of $AIC(D|S_{n})$ is reached at n=3 for $N\in \left\{16,\ldots ,128\right\}\cup \left\{512\right\}$, at n=4 for N=256, at n=7 for $N\in \left\{1024,\ldots ,8192\right\}$, and at n=8 for N=16,384. For the experimental range of sample size, the results suggest that, although the AIC score always elicits an EC cut point at n≥3, it is only equal to 3 for lower values of *N*. As *N* grows, the EC cut point also becomes larger. This is expected, as AIC penalizes less the complexity than MDL.

In order to examine the impact of the noise in the inferences drawn about the model selection criteria, similar experiments were performed on datasets with pNoise equal to 10% and 25%. Concerning the difference in entropy and the log-likelihood measures, the decreasing behavior is preserved, although the variation becomes less accentuated with noisier data. Moreover, since noise causes uncertainty, the jump from n=2 to n=3 is not expressive, and consequently, the early classification opportunity at n=3 is less obvious. For pNoise=10%, while the lowest value of MDL (Figure A3a) at n≥3 is attained for N≥256 (higher than for 5% noise), in AIC (Figure A3b) this minimum is reached for N≥32 (higher than for 5% noise).

Note that, in Figure A3, the curve N=4 also displays a minimum for $n\in \left\{2,\ldots ,4\right\}$. This event should not be considered relevant since the dataset is so reduced that noise has an unbalanced influence on the results. Proof of that is, for example, the curve N=8, which does not have a minimum at n≥3. For pNoise=25%, the lowest value of MDL (Figure A4a) at n≥3 is attained for N≥1024 (higher than for 10% noise), whereas in AIC (Figure A4b) it is reached for N≥64 (higher than for 10% noise). While Figure A3a (pNoise=10%) shows the MDL graph with some ambiguity in selecting the true model for larger values of *N*, Figure A4a (pNoise=25%) describes the same score identifying n=3 as the early time point with zero error. Furthermore, a lower deviation from the true distribution is also observed in AIC, for pNoise=25% (Figure A4b), in comparison with the case with pNoise=10% (Figure A3b).

A few considerations about the response of the MCEC method to variations on the size of the dataset can be referred to. Firstly, concerning the univariate context and for datasets with time-series of fixed length, the number of instances has a significant impact on both scoring functions and a not so strong influence in the difference in entropy and the log-likelihood measures. Besides, the results suggest there is a value of *N* from which the minimization of the model selection criteria is achieved at n=3. This indicates that the number of instances in a dataset influences the effectiveness of the scoring functions in selecting the actual distribution. It is well known that both model selection criteria are unsuitable for reduced datasets, where the number of instances is not considerably higher than the number of estimated model parameters, leading to overfitting.

Concerning the comparison between both criteria, the experiments demonstrate that, in general, AIC outperforms MDL, for more reduced datasets. However, for larger values of *N*, the AIC evidences a more significant deviation from the actual distribution, tending to choose more complex models than MDL. This fact verifies the MDL reputation of being more consistent than AIC in selecting the underlying model among the candidates, provided that the true model is in the set of alternatives.

In general, the sharp decreases in $H\left(C|A_{n}\right)-H\left(C|A,B\right)$ and in $LL(D|S_{n})$ for n=3, together with the minimum values depicted in both scores, give confidence in the early classification potential of the proposed method. On the other hand, the experiments demonstrate that the decision upon the early time point (*n*) is not always unanimous among the three measures that compose the MCEC algorithm. This means that, in some cases, the instant from which the remaining of the time-series in the dataset can be neglected is not uniquely identified.

Additional experiments were performed to the proposed method in order to analyze the impact of the variation of two other parameters: the number of features (*m*) and the time-series length (*L*). The objective consists of not only examine the early prediction opportunity, but also continue the investigation on how the size of the dataset influences the model selection criteria.

Seeing that the algorithm is capable of handling multivariate time-series (m≥2), the study involves randomly generated datasets with $m\in \left\{1,2,3,5\right\}$, while x=3, L=10 and $pNoise\in \left\{0\%,5\%\right\}$. With regard to the difference in entropy and the log-likelihood, the decreasing behavior of these measures is not substantially affected by the variation on the number of features per time point. In general, the reduction within $n\in \left\{2,\ldots ,4\right\}$ is expressive, which indicates that, in this time period, there occurs a significant decrease on the amount of information needed to predict the time-series classes of the dataset.

Considering the scoring functions, the value of *N* from which both criteria display a minimum at n≥3 increases with *m*. Table A1 confirms this inference by describing the variation on the number of instances for which the minimum of MDL and AIC is attained at n≥3, according to the number of features per time point.

**Table A1:** Values of *N* from which the scoring functions display a minimum at n≥3. Parameters: x=3, L=10, $pNoise\in \left\{0\%,5\%\right\}$ and $m\in \left\{1,2,3,5\right\}$.

*m*	pNoise	*N*	
		MDL	AIC
1	0%	32	16
	5%	128	16
2	0%	1024	128
	5%	2048	256
3	0%	8192	1024
	5%	32768	4096
5	0%	>131,072	65,536
	5%	>131,072	>131,072

In fact, for all experiments, the minima were reached for n=3. Moreover, AIC seems to be less dependent on *m* than MDL, since its values of *N* are always lower. This suggests that, although the dataset size impacts the effectiveness of the model selection criteria, the early classification time point is identified with reliable consistency.

Another parameter examined was the length of the time-series in the dataset. Although the proposed method requires the data to have a fixed *L*, this value can vary from database to database.

In order to evaluate how the variation of the time-series length affects the MCEC algorithm, several experiments were performed with $L\in \left\{6,10,18,38,78,158\right\}$, for the following fixed parameters: x=3, m=1 and $pNoise\in \left\{0\%,5\%\right\}$.

Concerning the curves from $H\left(C|A_{n}\right)-H\left(C|A,B\right)$ and $LL(D|S_{n})$, the impact of the variation of *L* is not significant. Table A2 includes the values of *N* from which both scoring functions show a minimum at n≥3.

**Table A2:** Values of *N* from which the scoring functions display a minimum at n≥3. Parameters: x=3, m=1, $pNoise\in \left\{0\%,5\%\right\}$ and $L\in \left\{6,10,18,38,78,158\right\}$.

*L*	pNoise	*N*	
		MDL	AIC
6	0%	32	16
	5%	64	32
10	0%	32	16
	5%	128	16
18	0%	32	16
	5%	128	16
38	0%	32	16
	5%	32	32
78	0%	32	16
	5%	64	32
158	0%	32	16
	5%	64	16

Unlike the results from Table A1, the lowest values of $MDL(D|S_{n})$ and $AIC(D|S_{n})$ were not consistently obtained for n=3, but instead, they deviated from the true distribution ($n\in \left\{4,6,7,8\right\}$) with the increase of the number of instances. The results demonstrate that the time-series length does not considerably condition the ability of both criteria to select the best model since the values of *N* in Table A2 do not significantly change with the variation of *L*. Although not always according to the expected model (n=3), and occasionally in a non-unanimous decision situation, the early classification opportunity is observable in the majority of the cases. One way of understanding this phenomenon is to notice that both the criteria are trying to model the noise itself by adding extra data points, which allows for correcting the noise.

In sum, these are the conclusions that can be drawn from the performed experiments based on the variation of the dataset size:

1. The number of instances (*N*) and the number of features per time point (*m*) have a significant impact on both model selection criteria and a not so strong influence in the difference in entropy and log-likelihood measures.
2. The time-series length (*L*) does not considerably affect none of the four measures.
3. As expected, with the increase of *m*, the number of instances (*N*) in a dataset also has to increase significantly for the method to select the true model, that is, to elicit the optimal early classification time point (*n*).
4. AIC is less dependent on *N* than MDL, but the latter identifies the true model more consistently than the first score.
5. The decision upon the early classification time point can be ambiguous; that is, the three main measures that compose the MCEC algorithm can propose distinct values of *n*.
